# The Golgi in Cell Migration: Regulation by Signal Transduction and Its Implications for Cancer Cell Metastasis

**DOI:** 10.1100/2012/498278

**Published:** 2012-05-01

**Authors:** Valentina Millarte, Hesso Farhan

**Affiliations:** ^1^Department of Biology, University of Konstanz, Universitätsstrasse 10, 78457 Konstanz, Germany; ^2^Biotechnology Institute Thurgau, Unterseestrasse 47, 8280 Kreuzlingen, Switzerland

## Abstract

Migration and invasion are fundamental features of metastatic cancer cells. The Golgi apparatus, an organelle involved in posttranslational modification and sorting of proteins, is widely accepted to regulate directional cell migration. In addition, mounting evidence suggests that the Golgi is a hub for different signaling pathways. In this paper we will give an overview on how polarized secretion and microtubule nucleation at the Golgi regulate directional cell migration. We will review different signaling pathways that signal to and from the Golgi. Finally, we will discuss how these signaling pathways regulate the role of the Golgi in cell migration and invasion. We propose that by identifying regulators of the Golgi, we might be able to uncover unappreciated modulators of cell migration. Uncovering the regulatory network that orchestrates cell migration is of fundamental importance for the development of new therapeutic strategies against cancer cell metastasis.

## 1. Introduction

The development of efficient therapies against cancer represents a continuous challenge for scientist across various disciplines. Among cancer-related death cases, 90% are due to metastasis formation. During metastatic dissemination, a tumor cell acquires a migratory phenotype that enables it to invade the surrounding tissue in order to leave the primary site of the tumor. After entering the blood or the lymph system, the tumor cell colonizes a distant tissue and nucleates a secondary tumor. Research in the past three decades has provided valuable insight into the various steps of tumor formation [1]. Nevertheless, the metastatic process remains poorly understood and our insight into the molecular events that initiate and/or sustain this process remains incomplete. This paper does not intend to give a general overview describing the metastatic process. For this, the reader is referred to excellent recent reviews [2]. In this paper we will focus on the role of the Golgi apparatus in cell migration and invasion and the implications thereof for cancer cell metastasis. We will first give a brief and general overview about the Golgi and about cell migration. Then, we will discuss in more detail the evidence that links the Golgi to cell migration. Finally, we will discuss how signaling pathways regulate the role of the Golgi in cell migration and how this knowledge can be used for designing novel therapeutic strategies against metastatic cancer cell spreading.

## 2. The Golgi Apparatus

In mammalian cells, the Golgi apparatus is a single-copy organelle, composed of a stack of flattened cisternae that are laterally linked to form the Golgi ribbon. The Golgi localizes to the juxtanuclear region and is intimately associated with the centrosome. The Golgi is polarized in both structure and function, where the *cis*- and the *trans*-side exert different roles in terms of posttranslational modification, lipid composition, and sorting events. The *cis*-Golgi receives cargo (proteins, lipids, and polysaccharides) from the ER and the intermediate compartment. Proteins that need to be recycled back to the ER are incorporated into COPI vesicles that form at the *cis*-Golgi. Cargo in the *cis*-Golgi then progresses to the *medial* and *trans*-Golgi where it acquires various post-translational modifications. Finally, cargo reaches the *trans-*Golgi network (TGN) for sorting to the various post-Golgi compartments (endosomes, lysosomes, and the plasma membrane). Besides posttranslational modification and sorting, the Golgi also plays important role in apoptosis, mitosis and cell migration. In this paper we will highlight the evidence for the role of signal transduction in regulating the Golgi during cell migration. A matter of hot and intense debate is how intra-Golgi transport is executed. According to the cisternal maturation model (alternatively referred to as cisternal progression), cargo remains in the same cisterna, which matures by acquisition and exclusion of different enzymes that convert the *cis*-cisterna to a medial and *trans*-cisterna. In an alternative model, the Golgi cisternae are stationary and cargo moves between them either by vesicular carriers or by tubular connections. Finally, a cisternal progenitor model was also recently proposed [3]. For detailed reviews about the functional organization of the Golgi and a general overview on signaling events at endomembranes, the reader is referred to other recent reviews [3–7].

## 3. Cell Migration

Cell migration is a dynamic and highly orchestrated cellular process. In a healthy organism, cell migration is involved in a variety of morphogenic events during organ and tissue development as well as in wound healing. Deregulation of migration is observed under several pathophysiological conditions such as inflammation and cancer. How a cell migrates depends mainly on the cell type, and for our review, we will concentrate on the migration of epithelial cells and fibroblasts. Directed migration of epithelial cells involves a complex cycle of leading edge protrusion, adhesion to the extracellular matrix, and retraction of the rear end of the cell accompanied by cycles of assembly and disassembly of actin filaments. Actin dynamics provide the major force for cell movement and actin polymerization at the plasma membrane drives protrusion formation. In order to form sheet-like protrusions (lamellipodia), actin forms branched filaments and for spike-like protrusion (filopodia) actin filaments are bundled. Actin dynamics at the plasma membrane are orchestrated by Rho-family GTPases. In addition, lipids and other proteins may provide spatial information and thereby contribute to overall actin regulation. Lamellipodia and filopodia are the main forms of protrusions when cells migrate on a 2D surface. However, migration in 3D involves two other types of protrusions called blebs and pseudopodia [8]. The ease of performing the assay is definitively an advantage of 2D cell migration studies. However, there are questions on the biologic relevance of findings derived from 2D migration studies. Nevertheless, most data on the role of the Golgi in cell migration are derived mainly from studies on 2D cell migration, and therefore, most of this paper will deal with 2D cell migration.

## 4. The Golgi Is a key Player in Cell Migration

Three decades ago, Kupfer et al. [9] studied the localization of the Golgi in experimentally induced wound (wound-scratch assay). They showed that the Golgi in cells remote from the wound exhibits a random orientation and is always positioned close to the nucleus. However, in cells located at the wound edge, the Golgi and the centrosome (also referred to as microtubule organizing center, MTOC) are both found facing towards the wound in the vast majority of cells. This paper marks the starting point for investigations on the role of the Golgi in directional cell movement, and until today, most studies report that the Golgi preferentially locates facing the leading edge. However, a recent study clearly showed that the position of the Golgi depends on geometrical constrains [10]. In a 2D scratch-wound assay, the leading edge is larger because the cells are released from constrains due to scratching. When cells were forced to migrate along a linear fibronectin pattern, the Golgi localized behind the nucleus, facing away from the leading edge [10]. However, as will be evident from the following, most data on the Golgi in cell migration are derived from scratch wound assays. More work will be needed to finally clarify the *in vivo* relevance of the findings on Golgi positioning in cell migration and whether the molecular events that we discuss in the next sections also apply to a Golgi that is positioned behind the nucleus. What is clear is that the Golgi does have an active role in cell migration, because various treatments that disrupted Golgi architecture were accompanied by an inhibition of cell migration. For instance, knockdown golgin-160 and GMAP210 led to fragmentation of the Golgi apparatus into many ministacks and also to an inhibition of cell migration [11]. This study relied on depletion of proteins that localize to the Golgi and regulate its structure. However, depletion of several kinases and phosphatases was also shown to alter Golgi structure and to inhibit directional cell migration [12, 13], which implies that the effect of some signaling pathways on migration is at least partially due to their effects on Golgi integrity. There are three important facts that relate to the role of the Golgi in cell migration: (i) the close association of the Golgi with the centrosome, (ii) polarization of secretory trafficking towards the leading edge, and (iii) orientation of microtubules towards the leading edge. Finally, we stress here that there is no clear evidence that the Golgi regulates cell migration *in vivo* (*i.e.,* in a living organism) and that most available evidence is based on cell culture experiments that were performed in 2D assays, that may not faithfully recapitulate the *in vivo* situation. 

### 4.1. MTOC and the Golgi

The work of Kupfer et al. [9] clearly showed that the orientation of the Golgi to the leading edge was coupled to the movement of the centrosome which implies a functional relationship between these two organelles. In a more recent work, it was shown that failure of the Golgi to orient towards the wound also blocks centrosome reorientation and this in consequence inhibits migration. Importantly, disassembling the Golgi by treatment with brefeldin A seemed to relief the block of MTOC movement and reallowed its orientation towards the wound [14], although migration was still inhibited. This result might tempt us to conclude that the role of the Golgi in cell migration is more prominent than that of the centrosome. This conclusion, however, is not completely correct, because it is based on treatment with brefeldin A, which blocks GDP exchange on Arf1, and therefore is expected to exert pleiotropic effects. Thus, more direct evidence was needed to get insight into possible differential roles for the Golgi and the centrosome in directional polarity. This would require unlinking the Golgi and the centrosome and this exactly is what Hurtado et al. achieved [15], who studied the centrosomal protein AKAP450 which also associates with the Golgi [16]. They used two different truncation-mutants of AKAP450 and overexpressed them in RPE-1 cells. Expression of a fragment termed AK1B led to a structural alteration of the Golgi, but the Golgi was still found at the centrosome. This AK1B fragment exerted only a mild inhibitory effect on directional cell migration and preserved the reorientation of the Golgi and the centrosome towards the leading edge [15]. Overexpression of a second fragment termed AK1 preserved the structural integrity of the Golgi but completely unlinked the Golgi from the centrosome. Under this condition, both the centrosome and the Golgi failed to reorient towards the leading edge and directional migration was strongly inhibited [15]. These findings are important because they clearly rank the association between the centrosome and the Golgi above the structural integrity of the Golgi. However, it has to be stressed here that the structural alteration of the Golgi by the AK1B fragment was very weak compared to what has been reported in the literature before. Therefore, the ranking of Golgi structure below its association with the centrosome has to be taken with caution.

### 4.2. Polarization of Secretory Trafficking

Despite the early evidence for the role of the Golgi and the MTOC in directional cell migration, it remained unclear why these organelles are important. The most obvious explanation is that orientation of the Golgi and the MTOC in the direction of migration facilitates polarized secretory trafficking towards the leading edge. Such a rapid and synchronized movement of the Golgi and the centrosome is also observed in other cellular processes such as the formation of the immunological synapse in natural killer cells and during formation and extension of the axon in hippocampal neurons [17]. Depletion of two Golgi peripheral proteins, GMAP210 and golgin-160, resulted in fragmentation of the Golgi and a loss of its pericentrosomal position accompanied by a significant impairment of polarized trafficking towards the leading edge [11]. Importantly, global trafficking between the endoplasmic reticulum and the plasma membrane was not affected. Thus, neither the structural integrity of the Golgi nor its pericentrosomal localization seems to be relevant for global secretory trafficking. The same conclusion has been reached by Hurtado et al. [15], who used fragments of AKAP450 that either affected Golgi integrity or displaced it from the centrosome. Under both conditions, global secretion was unaffected, but directed trafficking to the leading edge membrane was impaired [15].

### 4.3. Nucleation of Microtubules

**Figure 1:**
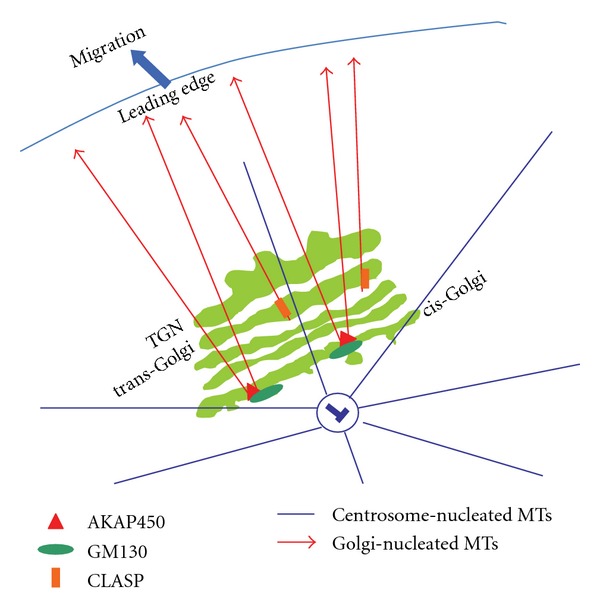
Schematic illustration of the Golgi in a migrating cell. The Golgi is oriented towards the leading edge and the microtubules (MTs) that nucleate from the Golgi are also oriented towards the leading edge (red), contrary to centrosomal MTs (blue) that are non-polarized.

The structure and the positioning of the Golgi are dependent on the microtubule (MT) cytoskeleton, which was first demonstrated by showing that the Golgi fragmented into several ministacks upon treatment with MT-depolymerizing drug nocodazole [18]. Several factors have been identified to control MT-dependent Golgi positioning which include small GTPases like Arf1 or Cdc42 and their regulators (such as ARHGAP21) [19]. In mammalian cells, microtubules were classically considered to nucleate at the centrosome, which was thought to be the sole microtubule organizing center. However, recent results from different groups demonstrated that the Golgi may serve as an additional nucleation platform for MTs (Figure 1). The notion that the Golgi is an MT-nucleating organelle was already raised a decade ago [20] when the occurrence of noncentrosomal MTs was observed in nocodazole-washout experiments. These additional MTs organizing units always colocalized with a Golgi ministack. In addition, purified Golgi membranes were shown to promote MT assembly. Importantly, it was shown that *γ*-tubulin was found to be associated with the Golgi and to be responsible for MT nucleation [20]. Later Ríos et al. showed that the peripheral Golgi protein GMAP210 mediates recruitment of *γ*-tubulin to the *cis*-Golgi and thereby was suggested to be a key player in MT nucleation at the Golgi [21]. It should be noted here that depletion of GMAP210 led to loss of the pericentrosomal Golgi localization and to an inhibition of directional cell migration [11]. Although the finding of Ríos et al. [21] clearly demonstrated an involvement of the Golgi in MT nucleation, it also raised questions about whether MT nucleate primarily on the Golgi, or whether they nucleate at the centrosome and are then released and captured by the Golgi. Another question that is provoked by these findings is whether Golgi-originating MTs are qualitatively or functionally distinct from those that nucleate at the Golgi? A first step to answer these questions was made by the discovery that CLASPs are required for formation of MTs at the Golgi [22]. CLASPs are plus-end MT binding proteins that were previously shown to localize to the Golgi [23]. CLASPs are recruited to the trans-Golgi network (TGN) in a manner dependent on GCC185, a mammalian GRIP domain golgin protein. Depletion of GCC185 led to dispersion of CLASPs from the Golgi but did not affect MT plus-end binding [22]. Importantly, MTs originating from the Golgi were radial as those emanating from the centrosome. Instead, Golgi-nucleated MTs preferentially oriented towards the leading edge in migrating cells, thus contributing to the asymmetry of the microtubule network [22]. It is important to note here that CLASPs were shown to mediate MT nucleation at the TGN, but earlier reports clearly indicated a role of the *cis*-Golgi in MT biogenesis [21]. This raises the question on whether the *cis*-Golgi also bears proteins that are involved in MT nucleation in a manner analogous to CLASP. This question was (at least partially) answered by the finding that the *cis*-Golgi protein GM130 is involved in MT formation [24]. GM130 binds to AKAP450, a centrosomal protein, and thereby mediates MT biogenesis from the *cis*-Golgi. When the Golgi was disassembled by treatment with brefeldin A, AKAP450 redistributed to ER exit sites and MTs seemed to nucleate from this location, indicating that AKAP450 is necessary and sufficient for MT nucleation. Again, Golgi-emanating MTs were oriented towards the leading edge in migrating cells, and consequently, depletion of AKAP450 inhibited directional cell migration [24]. The studies discussed previously clearly establish that the Golgi is an MT-nucleating organelle. However, they do not take the nature of the Golgi into account. In case the Golgi is viewed as a collection of stable cisternae (see above), then one has to assume that two MT nucleation centers exist, with one at the *cis*-Golgi (controlled by GM130 and AKAP450) and the other at the TGN (controlled by CLASPs). Alternatively, if we assume that the cisternal maturation model is correct, MTs forming at the *cis*-Golgi in an AKAP450-dependent manner will move on as the cisterna is maturing. However at the medial to *trans*-Golgi, there is no GM130 and thus not AKAP450 to capture the MTs. Thus, MTs have to be anchored to the cisterna in an AKAP450-independent manner. Alternatively, the MTs formed at the *cis-*Golgi are handed over as the cisterna matures. In this scenario, CLASPs could serve to capture and/or stabilize MTs arriving at the TGN. This scenario requires the existence of other factors at the medial to *trans*-Golgi that are required for the formation of MTs. Whether these assumptions are true has to be determined in the future.

## 5. Signaling at the Golgi: Implications for Cell Migration

### 5.1. The Mitogen Activates Protein Kinase (MAPK) Pathway

**Figure 2:**
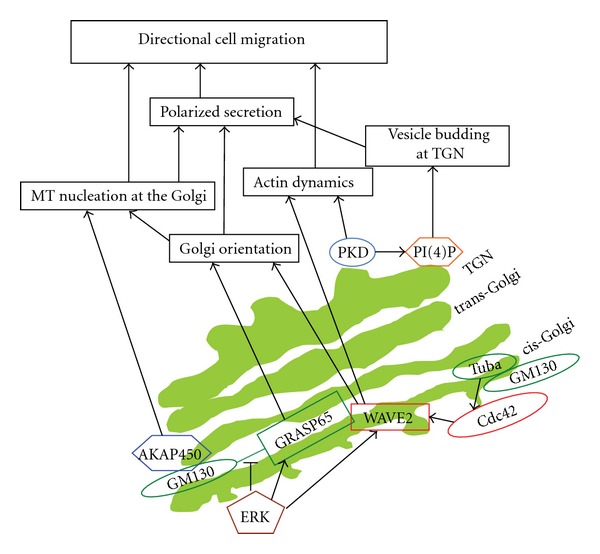
Schematic illustration of signaling events at the Golgi that regulate directional cell migration as explained in the main text.

The three main MAPK pathways are the extracellular signal regulated kinases (ERK1/2), the Jun N-terminal kinases (JNKs), and the p38 MAPKs. ERKs transmit signals downstream of a plethora of receptors and thus they orchestrate a wide range of biological processes such as proliferation, differentiation, and cell movement. Receptor signaling is transmitted to ERK1/2 via Ras GTPases, which are known to signal at the Golgi apparatus [6, 25, 26], although some recent evidence partially argued against this notion [27]. However, whether Golgi-localized active Ras has any direct role in cell migration remains to be determined. This appears likely to be the case, because downstream targets of Ras (in particular ERK) have been shown to regulate directional cell movement. The clearest example for an effect of ERK on the Golgi was provided in the work of Bisel et al. [14] who showed that ERK directly phosphorylates the Golgi matrix protein GRASP65 (Figure 2). When cells were stimulated with mitogens, GRASP65 was phosphorylated on serine277. Using an *in vitro* assay, the authors showed that phosphorylation of GRASP65 by ERK led to Golgi unstacking, a phenomenon not observed in intact cells. Intriguingly, serine277 is also phosphorylated by cdk1 during mitosis [28]. Mutation of serine277 to alanine led to an inhibition of Golgi (and centrosome) orientation towards the leading edge [14]. In conventional light microscopy, treatment of cells with mitogens does not change Golgi morphology ([14], our unpublished observations). Whether there is a mild rearrangement of Golgi structure which facilitates its orientation towards the leading edge remains to be determined. Phosphorylation of GRASP65 by ERK is a promigratory signaling event, but there is evidence that excessive ERK signaling might elicit inhibitory effects on migration. Cells that lack p190RasGAP, a Ras inactivating protein, display higher levels of active ERK but migration is inhibited in these cells [29]. Importantly, these cells also display a fragmented Golgi, which most likely accounts for the observed inhibition of migration [29]. These data can be interpreted as follows: the excessive Ras (and in consequence ERK) activation in p190RasGAP depleted cells results in fragmentation of the Golgi which in consequence inhibits directional cell migration. Another Ras modulator that was shown to play a role in cell migration is RasGRP1. In human T-lymphocytes, activation of the chemokine receptor CXCR4 led to translocation of RasGRP1 to the Golgi where it activated N-Ras and thereby ERK, and this was required for migration of these lymphocytes [30]. Whether an analogous effect for a Ras GEF exists in epithelial cells remains to be determined.

The Raf kinase inhibitory protein 1 (RKIP1) has been identified to act as a suppressor of metastasis in a variety of cancer types [31, 32]. RKIP1 overexpression inhibits cell migration and reduces the invasive potential of cancer cells. On the other hand, RKIP1 expression is reduced in specimens derived from metastatic lesions, thus emphasizing its role as a prometastatic protein [33]. RKIP1 inhibits ERK signaling by binding to and inhibiting Raf kinases, which are upstream activators of ERK. Loss of RKIP1 increases ERK signaling, and this is a condition that is thought to causally underlie the increase in cell migration and the elevated metastatic potential of the cell. It remains to be determined whether this is the case and whether this involves signaling to or from the Golgi.

Another regulator of ERK signaling is a protein called RKTG (Raf Kinase Trapping to Golgi; also referred to as Progestin and adipoQ receptor family member III, PAQR3). RKTG is a seven-transmembrane protein that localizes to the Golgi with its N-terminus facing the cytosol [34]. RKTG binds to Raf kinases and sequesters them to the Golgi and in consequence inhibits ERK signaling [35]. Despite the well known connections of Golgi and migration on one side and ERK signaling and migration on the other side, very little is known about whether RKTG interferes with ERK signaling to the Golgi in the context of cell motility. Recently, RKTG was shown (by negatively regulating ERK signaling) to inhibit hypoxia-inducible factor-1*α* (HIF-1*α*) and to suppress VEGF transcription, thereby reducing hypoxia-induced VEGF production [35]. Thus, RKTG is involved in regulating tumor angiogenesis [35]. However, whether RKTG has a direct impact on the motility of epithelial cancer cells remains to be determined in the future. 

From all MAPKs, ERK is the best characterized in terms of its role at the Golgi. Although members of the JNK and the p38 MAPK family have well-appreciated roles in cell migration, a clear link of these kinases to Golgi is missing. Our previous work showed that knockdown of JNK2, JNK3 and p38alpha MAPK disrupts Golgi architecture and inhibits migration [12], therefore tempting to speculate about a role of these MAPKs in regulating the Golgi in cell migration. JNKs have been shown to signal downstream of Rho family GTPases, which are well known regulators of cell migration (see the following). MEKK4 was shown to localize to the Golgi, to interact with Rac1 and Cdc42, and to mediate signaling of these GTPases to JNK [36]. Also MLK3 localizes to the Golgi and mediates Cdc42 signaling to JNK [37]. These findings offer a framework for future investigations on a potential role of JNK and p38 MAPK signaling from the Golgi in cell migration.

### 5.2. Rho Family GTPases

Rho family GTPases are small G-proteins consisting of three main members, Rho, Rac, and Cdc42, which were shown to regulate a variety of cellular processes such as cell death, phagocytosis, and cell polarity. As with all G-proteins, Rho family GTPases cycle between a GDP (inactive) and a GTP (active) bound state. In addition, while inactive Rho family GTPases are bound to Rho-GDI (guanine-nucleotide dissociation inhibitor) that functions as a chaperone preventing nonspecific, premature activation.

In a wound scratch assay, activation of Cdc42 has been observed to occur within the first hour after wounding suggesting that Cdc42 activation is an early event in cell migration [38]. In agreement with this, depletion of Cdc42 inhibited directional cell migration. However, although the involvement of Cdc42 in directional polarity is beyond any doubt, major evidence came from biochemical experiments that do not allow any conclusion about spatial aspects of this activity. Therefore, considerable efforts in the past 10 years were spent on the development of fluorescent sensors that allow visualization of Cdc42 (and other Rho GTPase members) activity in living cells. These reporters are mostly based on FRET (fluorescence resonance energy transfer), and a review on the various constructs that were designed to report spatiotemporal signaling of Rho family GTPase members has been published very recently elsewhere [39]. Using these fluorescent reporters, several groups reported on the existence of a pool of Cdc42 at the Golgi [40, 41]. However, most of these experiments are based on overexpressed Cdc42, which might lead to oversaturation of Rho-GDI resulting in a higher basal activity of Cdc42 in these cells, and therefore, more work is needed to elucidate whether Cdc42 is truly active at the Golgi. In addition, it has to be determined whether active Cdc42 that is observed at the Golgi is the result of local activation or whether Cdc42 was activated at another location and is then transported in its active form to the Golgi. A recent report suggested yet another role for the Golgi in controlling Cdc42 activity. There, Osmani et al. investigated mechanisms controlling the localized and restricted activation of Cdc42 at the plasma membrane [42]. They showed that enrichment of Cdc42 was dependent on post-Golgi membrane trafficking. This finding indicates that the role of the Golgi in controlling Cdc42 activity is mediated by directing secretory traffic towards the leading edge, supplying the plasma membrane with modulators of Cdc42 activity [42]. 

In support for a *bona fide* activity of Cdc42 at the Golgi was the discovery that the Golgi matrix protein GM130 interacts with Tuba and recruits it to the Golgi [43]. Tuba is an exchange factor for Cdc42, and therefore, this discovery would indicate that Cdc42 is activated locally at the Golgi. This was further supported by the finding that knockdown of GM130 by siRNA reduced the steady-state level of active Cdc42 [43]. However, it should be noted here that cell migration is unlikely to be mediates by the steady-state levels of active Cdc42, but rather the result of induced Cdc42 activity and this was not tested for. In addition, the pool of Tuba observed on the Golgi in this report was very faint and most other findings (including our own unpublished observations) indicate that Tuba primarily localizes to locations other than the Golgi [44]. Therefore, more work is needed to determine whether Golgi membranes provide an environment where Cdc42 can be activated. This is important in order to support a primary role for the Golgi in directional cell migration where the Golgi not only responds to polarity signaling originating from the plasma membrane, but in fact initiates such polarity signaling. Further attempts will also have to focus on whether active Cdc42 at the Golgi regulates other signaling pathways involved in cell migration. For instance, does local Cdc42 activate atypical PKC at the Golgi and does this in turn induce the nucleation of MTs from the Golgi? This and many other questions will have to be answered in the future.

Like Cdc42, also some of its downstream effectors have been shown to localize to the Golgi and to be involved in cell migration. Signals from surface receptors through Rac and Cdc42 regulate the activity of the Wiskott-Aldrich syndrome protein (WASP) family and consequentially actin filament severing by binding to the Arp2/3 complex. Several members of the WASP family exist [45, 46] and include WASP (limited to the hematopoietic system) and N-WASP (ubiquitous). Furthermore, the WASP family includes the WASP and verprolin-homologous protein (WAVE)1 and 3 (both expressed exclusively in the brain), WAVE2 (ubiquitous), the newly identified WASP and SCAR homologue (WASH), WASP homologue associated with actin, membranes and microtubules (WHAMM), and junction-mediating and regulatory protein (JMY). Using cryoimmunoelectron microscopy, Cdc42 and its effectors N-WASP and Arp2/3 were found to localize to the Golgi complex [47]. This raises the question of whether WASPs and the Arp2/3 complex are involved in cell migration. Early evidence suggested that this might be the case [48], and it was clearly proposed that WASPs would promote cell migration and invasion (and therefore be prometastatic). However, recent findings call for a more differentiated view on the role of WASPs in cancer cell metastasis, because depending on the cell/tissue type, WASPs may exert either a positive or negative effect on migration and invasion [46]. For instance, the same work showed that while WAVE2 was involved in Golgi polarization in NIH3T3 cells, it had no appreciable role in Golgi polarity in astrocytes [48]. WASP proteins have been shown to be regulated by signaling. For instance, in NIH3T3 cells, the supportive role of WAVE2 in Golgi polarization and cell migration is regulated by signaling by the ERK cascade, a signaling pathway that is well-known to regulate cell migration. Overexpression of a WAVE2 mutant that cannot be phosphorylated inhibits orientation of the Golgi towards the leading edge and in consequence also directional cell migration [49]. This example nicely illustrates how two regulators of cell migration, namely, WAVE2 and ERK, may exert their roles cooperatively. The promigratory effect of ERK was also ascribed to phosphorylation of GRASP65 [14]. Therefore we might ask whether phosphorylation of GRASP65 and WAVE2 by ERK occurs simultaneously, and if yes, whether there is any dominance of one effect over the other and finally we have to determine whether these effects are cell-type specific in order to reach a generally applicable conclusion that would qualify these molecular events to be potential targets for antimetastatic cancer therapy. Phosphorylation of WAVE2 in the context of cell migration has been shown by several kinases such as Cyclin-dependent kinase 5 [50] or Casein kinase-2 [51]. Whether these events occur on the Golgi remains to be determined. Targeting Rho signaling is important in light of the fact that upstream regulators as well as downstream signaling molecules of Rho GTPases were shown to determine the metastatic potential of several cancer types. For instance, PAK1, which signals downstream of Cdc42 and Rac, was shown to be overexpressed in hepatocellular carcinoma and expression of PAK1 was furthermore shown to correlate with the metastatic potential of this cancer type [52]. For other proteins linked to Rho GTPases, the situation is less clear. Divergent findings have been made concerning the role of RhoGDI2 in cancer, which seems to depend on the type of cancer investigated. For instance, in ovarian cancer, RhoGDI2 was shown to be a suppressor of proliferation, invasion, and metastasis [53]. RhoGDI2 expression is elevated in gastric cancer [54] but downregulated in bladder cancer [55]. These two examples (PAK1 and RhoGDI2) illustrate the importance of Rho GTPases in determining the metastatic potential of cancer cells.

### 5.3. Cyclin-Dependent Kinases

Upon entry into mitosis, the Golgi apparatus disassembles and reforms at the end of mitosis in the two new daughter cells [56–58]. Therefore, it is not surprising that Golgi proteins were shown to be phosphorylated by mitotic kinases such cyclin-dependent kinases (Cdk). Apart from mitotic effects, there are atypical Cdk family members that are activated by proteins other than cyclins and Cdk5 is one of these [59]. Cdk5 was shown to phosphorylate the Golgi protein GM130 and thereby to mediate Golgi fragmentation in apoptotic neurons [56]. Cdk5 has a well-documented role in cell migration where the majority of effects described are due to alterations of the actin cytoskeleton via modulating proteins like p190RhoGAP, focal adhesion kinase, and ROCK [59, 60]. All migration-related effects of Cdk5 were described for effects that are unrelated to the Golgi. However, Cdk5 has been shown to not only localize to the Golgi but also to interact with Cdc42 and to phosphorylate the Cdc42 effector PAK1, and this phosphorylation was suggested to play a role in formation of Golgi carriers [61]. This study has been performed in neurons, but it certainly tempts to speculate about a potential role of Golgi localized Cdk5 in the context of epithelial cell migration.

### 5.4. Phosphoinositide Signaling

There are seven distinct phosphoinositide subspecies that are involved in a wide range of biological effects such as proliferation, differentiation, vesicle trafficking, and cell survival. Among the different phosphoinositides, phosphatidylinositol-4-phosphate (PI(4)P) formed by different PI-4-kinases (PI4K) is most important for the regulation of various biological functions of the Golgi. In mammalian cells, PI4KII*α* and PI4KIII*β* account for the synthesis of the PI(4)P pool on the Golgi [62]. At the trans-Golgi, the PI(4)P rich membrane serves as a docking platform for GOLPH3 (also known as GPP34 or yeast Vps74p) [63, 64]. GOLPH3 interacts with MYO18A and thereby links the Golgi to the actin cytoskeleton, which gives the Golgi its extended morphology [64]. GOLPH3 is also involved in budding of carriers at the *trans*-Golgi. Therefore, GOLPH3 is a PI4K effector protein that mediates effects on Golgi morphology and trafficking. Interestingly, GOLPH3 was shown to regulate mTOR (mammalian target of rapamycin) signaling [65], a finding that links PI4K signaling at the Golgi to the regulation of cell growth. A possible explanation is that GOLPH3 interacts with VPS35, a component of the retromer complex which is known to regulate endocytic trafficking transmembrane receptors [66]. A direct link between PI(4)P at the Golgi and the role of this organelle in cell migration does not exist. However, GOLPH3 could provide a potential link in the future as it regulates mTOR signaling, which regulates cell migration (see the following). In addition, GOLPH3 regulates endocytic trafficking of transmembrane receptors which have a role in cell migration. Finally, GOLPH3 regulates budding of vesicles from the Golgi, and polarization of secretory trafficking is one of the major factors accounting to the role of the Golgi in directional polarity (Figure 2). However, at the current stage this remains speculative and future experiments will have to address the question whether GOLPH3 plays any role in cell migration. While a promigratory role of GOLPH3 remains speculation, in the next section we will discuss an example how a regulator of budding at the TGN was shown to be involved in cell migration.

### 5.5. Protein Kinase D Signaling

Protein kinases D1-3 (PKD1-3) were initially classified as diacylglycerol-stimulated members of the protein kinase C family of serine-threonine kinases but, due to limited similarity, were reclassified as a novel group of the calmodulin-dependent kinase family [67]. PKD has been found over a decade ago to localize to the Golgi and to control the budding of secretory cargo at the TGN towards post-Golgi compartments in a manner dependent on phosphorylation and activation of PI4KIII*β* [68–70]. As mentioned previously, one of the roles of the Golgi in directional cell migration is due to the polarization of secretory cargo towards the leading edge. In accordance with this, inhibition of PKD, by using a kinase-dead version, was shown to inhibit formation of lamellipodia at restricted areas of the cell surface and thus random cell motility was strongly affected [71]. This work provided the first evidence on a potential role of PKD in cell migration, but its role in directional migration was not tested. Later it was found that dominant negative PKD as well as PKD knockdown by siRNA actually increased directional cell migration [72]. The inhibitory effect of PKD on cell migration is mediated in part by phosphorylation of cortactin [73], an actin-binding protein enriched in lamellipodia of motile cells and invadopodia of invasive cancer cells [74]. Furthermore, PKD was shown to phosphorylate the slingshot homologue 1 (SSH1), which results in its cytosolic sequestration [75, 76]. PKD-mediated phosphorylation of SSH1 blocks it from activation of the actin depolymerizing factor cofilin. As a consequence loss of PKD induces migration. Thus, PKD is an excellent drug target for antimetastatic cancer therapy, where activators of PKD are expected to act as suppressors of metastasis. Such an approach is currently being tested using the macrolactone Bryostatin 1, which activates PKD1 [77] and is currently in phase-II clinical trials where its antineoplastic effects are being evaluated. 

### 5.6. mTOR Signaling

Mammalian target of rapamycin (mTOR) is a serine-threonine kinase that belongs to the family of phosphatidylinositol kinase-related kinase (PIKK family). mTOR exists in two distinct multiprotein complexes, the rapamycin-sensitive TORC1 (composed of mTOR, Raptor, mLST8, PRAS40, Deptor) and the rapamycin-insensitive TORC2 (composed of mTOR, Rictor, mLST8, mSIN1, Protor, Deptor). TORC1 has a well-appreciated role in the regulation of cell growth, and metabolism [78] and many upstream signaling pathways have been identified to control TORC1 activity, including, for example, ERK1/2, Akt, and GSK3*β* (for a comprehensive list see [79]). In contrast to TORC1, the pathways leading to TORC2 activation are ill defined, but recent evidence suggests that PI3K signaling, ribosomes, and Rac1 may activate TORC2 [80–82]. Both mTOR complexes were shown to be involved in cell migration. TORC1 has been shown to localize the actin arc (at the leading edge) in migrating cells, where it activates p70S6 kinase and thereby controls actin dynamics [83]. Inhibition of TORC1 by rapamycin resulted in an inhibition of cell migration [83]. Rapamycin treatment also activated PP2A and in consequence to this inhibited IGF1-induced cell movement [84]. 4E-BP1, a translation initiation factor, is a known downstream target of TORC1 and was also shown to contribute to the promigratory role of TORC1. It was shown that inhibition of TORC1 by rapamycin reduced the activity of 4eBP1 and reduced the expression of RhoA, Rac1, and Cdc42, which are all important migration regulators [85]. Also TORC2 has been shown to regulate cell migration, which was to be expected given that the first biological effect ascribed to TORC2 was the regulation of the actin cytoskeleton via activation of Rho GTPases in yeast and in human cells [86, 87]. Indeed, chemotaxis of neutrophils has been shown to be regulated by TORC2 dependent activation of RhoA [88]. For a comprehensive review on the role of TORC1/2 in cell migration, the reader is referred to a recently published review [79]. All these effects were mostly linked to events at the leading edge plasma membrane. However, there is evidence that would suggest investigating potential roles of TORC1/2 at the Golgi in the context of cell migration. mTOR was shown to target to the Golgi via its HEAT repeat domain [89]. There is evidence that TOR can be activated at the Golgi because (in yeast) Golgi-localized Mn^2+^-ATPase is capable of regulating TOR signaling [90]. Upon infection of mammalian cells with *Toxoplama gondii*, the centrosome and the Golgi relocate into close proximity of the parasitophorous vacuole and this movement of the Golgi and the centrosome is dependent on mTORC2 [91]. Therefore, it seems worthwhile to investigate whether mTORC1 and mTORC2 signal from the Golgi and thereby regulate the machinery involved in directional cell migration.

## 6. Concluding Remark

The secretory pathway and particularly the Golgi are increasingly being viewed as hubs for signaling molecules contributing to the spatial organization of several signal transduction pathways. The Golgi apparatus plays a well-appreciated role in cell migration and invasion. As highlighted in this paper, there are several known as well as many potential signaling pathways that regulate the Golgi in the context of cell migration. Migration and invasion represent the cell biological basis for the metastatic dissemination of tumor cells, and therefore, any inhibitor of cell migration represents a potential antimetastatic drug. However, we are only beginning to grasp the extent of the regulation of the secretory pathway by signaling. Research in the future has to concentrate on uncovering the full regulatory network that orchestrates the Golgi during cell migration. We have recently found 38 kinases and phosphatases that when depleted lead to Golgi fragmentation and inhibition of migration [12]. Only 10% of our hits overlap with the hits from another RNAi screen on cell migration [92]. We propose a “Golgi-centric” strategy for discovering new therapeutic drug targets that regulate cell migration. This approach can be used as a complementary strategy to previous work that screened siRNA libraries or chemical compound libraries for effects on migration and invasion [92, 93] to uncover novel targets for antimetastatic cancer therapy.
